# Hepatoprotective effect of Qushihuayu formula on non-alcoholic steatohepatitis induced by MCD diet in rat

**DOI:** 10.1186/s13020-021-00434-1

**Published:** 2021-03-16

**Authors:** Qingping Lan, Zhitao Ren, Yan Chen, Guozhen Cui, I. Cheong Choi, Carolina Oi Lam Ung, Hon Ho Yu, Simon Ming-Yuen Lee

**Affiliations:** 1 State Key Laboratory of Quality Research in Chinese Medicine and Institute of Chinese Medical Sciences, University of Macau, Macao, China; 2 Zun Yi Medical University- Zhuhai Campus, Zhuhai, China; 3 Department of Gastroenterology, Kiang Wu Hospital, Macao, China; 4 Institute of Chinese Medical Sciences, University of Macau, Room 7003, N22 Building, Avenide da Universidade, Taipa, Macau, China

**Keywords:** Non-alcoholic fatty liver disease, Non-alcoholic steatohepatitis, Traditional Chinese medicine, Qushihuayu, MAPK pathway, PPAR-γ, P-p65, Fibrosis, Inflammation, HSCs reprogramming, Methionine-choline deficient diet

## Abstract

**Background:**

Non-alcoholic steatohepatitis (NASH) is an advanced form of non-alcoholic fatty liver disease (NAFLD) for which there is yet any standard pharmacotherapy. Traditional Chinese medicine formula such as Qushihuayu (QSHY) composing of multiple bioactive compounds has been used to treat NAFLD and NASH and shows beneficial effects over single compound treatment. This study aimed to investigate the mechanism of hepatoprotective effect of QSHY formula using a rat model.

**Methods:**

Six-weeks old male Wistar rats were given methionine/choline supplemented (MCS) diet for 8 weeks and used as the blank control. Another 7 rats, which received methionine/choline deficient (MCD) diet in the first 6 weeks and a MCS&MCD (1:1) mixture diet in the last 2 weeks, were used as the model group. The groups of QSHY pre-treatment, low dosage, medium dosage and high dosage were given the same diet as the model group. Except for pre-treatment group (1 week in advanced of other groups), all QSHY treatment groups received QSHY formula by gavage every day since the MCD diet started.

**Results:**

In the MCD diet group, the QSHY formula decreased the serum ALT and AST levels, lipid droplets, inflammation foci, FAS and α-SMA protein expression than MCD diet group. MAPK pathways phospharylation were markedly depressed by the QSHY formula. Moreover, QSHY formula enhanced PPAR-γ and p-p65 translocating into nucleus. The administration of QSHY increased hepatic mRNA levels of Transcription Factor 1 alpha (HNF1A), Hepatocyte Nuclear Factor 4 alpha (HNF4A) and Forkhead box protein A3 (FOXA3) which play a pivotal role in Hepatic stellate cell (HSCs) reprogramming.

**Conclusion:**

These findings suggest that QSHY formula exerts a hepatoprotective effect against steatosis and fibrosis presumably via depressed MAPK pathways phosphorylation, reinforcement of PPAR-γ and p-p65 translocating into nucleus and enhanced HSCs reprogramming.

**Supplementary Information:**

The online version contains supplementary material available at 10.1186/s13020-021-00434-1.

## Background

Non-alcoholic fatty liver disease (NAFLD) is a common cause of liver disease with a high prevalence worldwide that continues to grow [1]. NAFLD represents a spectrum of histological abnormalities that ranges from simple fatty liver to the more advanced form of non-alcoholic steatohepatitis (NASH) featuring insulin resistance. NAFLD shares common pathophysiology with other metabolic syndrome that may explain why they frequently coexist. The patients who suffered from obesity and type II diabetes have been shown to carry a higher risk of NAFLD [2]. Although most of NAFLD patients have simple steatosis, 7–30% of the cases developed chronic hepatic inflammation associated with cirrhosis, portal hypertension and hepatocellular carcinoma. At present, the causes of progression from NAFLD to NASH remain unclear.

Traditional Chinese Medicine (TCM) has been widely studied for the management of the root cause of NASH, i.e. fatty liver [3]. According to the TCM theory, fatty liver is featured with deficiency of three Zang viscera including the liver, spleen and kidney, resulting in patients’ symptoms of Qi deficiency, liver and kidney deficiency, phlegm and dampness heaping internally, and Qi stagnation and blood stasis [4]. The TCM treatment was mainly to resume the balance by nourishing Qi, strengthening the liver, clearing heat, discharging phlegm, and rejuvenating the blood [5]. A large number of clinical trials reported positive effects of herbal medicines involving TCM herbs on serum aspartate aminotransferase, alanine aminotransferase, glutamyl transferase, alkaline phosphatases, ultrasound, and computed tomography scan [6]. Positive effects of the herbal medicines on the recovery rate, total effective rate, and liver function without the risks of serious adverse reactions as compared with conventional medicines were reported [5]. n particular, TCM treatment has been shown to improve NASH through the modulating effect of insulin resistance, which is a key factor that plays an important role in the development of the serious liver condition [7].

Qushihuayu (QSHY), a TCM formula developed for fatty liver disease and used in clinical practice to treat NAFLD in China for more than a decade, has also been studied for its effect on NASH. The QSHY formula composed of 5 herbs (*Curcuma longa L.*, *Artemisia capillaris Thunb, Gardenia jasminoides*, *Hypericum japonicum Thunb.ex Murry*, and *Polygonum cuspidatum Sieb.et ZuccO*. *Curcuma longa L* exhibited antioxidant, hepatoprotective, antisteatotic, and anti-inflammatory, effects by regulating apoptosis, CYP2E1, Nrf, lipid metabolism-related factors, TGF-*β*, NF-*κ*B, CYP7A1, and so on [8]. *Artemisia capillaris Thunb*, aqueous extraction, have effect on lipopolysaccharide-induced inflammatory response by preventing NF-κB activation in rat liver [9]. *Gardenia jasminoides* also have hepatoprotective effect in bile duct ligated rats and hepatic stellate cells [10]. Total aqueous extract of *Hypericum japonicum* can decrease AST and ALT levels in serum in CCl4-induced liver injury in mice [11]. Huzhang, the root of *Polygonum cuspidatum Sieb. et Zucc.,* is widely used in anti-cancer, anti-inflammatory and anti-oxidative due to the major bioactive compound resveratrol and polydatin [12].

Current studies have focused on identifying the chemical basis of QSHY formula responsible for the anti-NAFLD and anit-NASH effects, and exploring the mechanisms of actions. Research on the chemical basis had identified curcuminoids and sesquiterpenoids as the key components of QSHY formula, both of which were known to have anti-oxidative, anti-carcinogenic, and anti-inflammatory activities collectively. QSHY formula used in Feng’s research showed effect on improving hepatic anti-oxidative, decreasing hepatic lipid synthesis, and promoting the regulatory T cell [13]. The study by Nishiyama et al. indicated that both curcuminnoids and sesquiterpenoids exhibited hypoglycemic effects via PPAR-gamma activation as one of the mechanisms of action [14]. A more recent study profiled the ingredients of QSHY formula and their metabolism in vivo and in vitro and identified 66 constituents identified in the QSHY formula and 34 metabolites through eight metabolic pathways [15].

Metabolomic studies have been employed to determine the possible mechanism of action of QSHY formula. For instance, Gou et la employed serum and liver tissue metabolomics approach and found that QSHY formula had significant anti-fatty liver effects through regulating the dysfunction of beta-alanine metabolism, alanine, aspartate, and glutamate metabolism, glycine, serine, and threonine metabolism, pyruvate metabolism, and citrate cycle [16]. Another metabolomic study even suggested that QSHY formula might be able to prevent fatty liver through regulating the dysfunctions of phenylalanine, tyrosine, tryptophan biosynthesis, phenylalanine metabolism, and tryptophan metabolism [17]. QSHY formula was also suggested to exhibit significant effect on inhibiting hepatic lipid accumulation via AMPK pathway [18]. Another study proposed that QSHY formula simultaneously enhanced the hepatic anti-oxidative mechanism, decreased hepatic lipid synthesis, and promoted the regulatory T cell inducing microbiota in the gut [13].

At present, the bioactive constituents responsible for the efficacy of QSHY formula and the mechanisms of action for NASH have not been fully clarified, warranting further research to improve the current understanding about the potentials of QSHY formula in the treatment of NASH. Therefore, this study aimed to investigate the hepatoprotective effect of QSHY formula in NAFLD and NASH by employing a mice model.

## Methodology

### Rat Model

Major approaches to NASH induction can be classified as follows: (1) Genetic approach; (2) nutritional approach; and (3) a combination of genetic factors with others such as nutritional factors, oxidative stress, and drugs [19]. Methionine/choline deficient (MCD) diet is one of the most widely used methods to build NASH model. As early as 3 days after the start of MCD diet, the mice may develop hepatic inflammation. Severe pericentral steatosis may occur by 1–2 weeks and necroinflammation may develop after 2 weeks. Oxidative stress can be observed from 3 weeks post MCD diet. However, the development and severity of MCD-induced NASH in rodents may depend on the gender, strain, and species used. It is slower in Sprague–Dawley rats than in other rodents to develop steatohepatitis induced by MCD diet. The MCD diet induced NASH model is one of the best established models to study the evolution of inflammation, oxidant stress and fibrotic changes because this model is easily established and severer than other nutritional models [20].

### Animal

6 weeks-old male Wistar rats (200–280 g), were housed on 12 h light/12 h dark cycling. The environment was well controlled for temperature and humidity and free access to food and water was available. Rats were given either MCD diet (L-amino acids 175.7 g/kg, sucrose 441.9 g/kg, cornstarch 15.0.0 g/kg, dextral maltose 55.0 g/kg, cellulose 30.3 g/kg, corn oil 100.0 g/kg, sodium hydrogen carbonate 7.4 g/kg, salt mixture35.0 g/kg, vitamin mixture 10.0 g/kg) or methionine-choline- supplemented (MCS) diet (choline 2 g/kg, methionine 3 g/kg) as normal diet. MCD diet and MCS diet were supplied by Trophic Animal Feed High-Tech Co., Ltd., according to the protocols approved by Animal Research Ethics Committee of University of Macau (UMARE-001–2017). All rats were divided into 7 groups randomly (Table 1). Food intake were measured every week. All rats were killed for blood collection and tissue sampling after 8 weeks treatment.

**Table 1:**
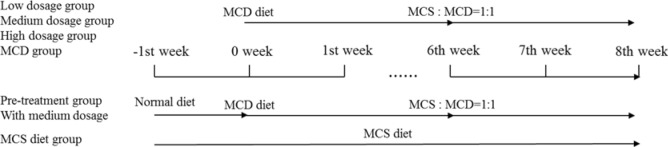
Animal groups

6 weeks old male Wistar rats were divided into 7 groups randomly. Low dosage group, medium dosage group, high dosage group, MCD diet group and pre-treatment group were received MCD diet in the first 6 week and then the diet was changed to the mixture of MCS diet and MCD diet with the ratio of 1:1. Pre-treatment group were received the medium dosage QSHY formula in advance one week before MCD diet treatment. Other drug treatment groups were received the drug treatment when the MCD diet treatment started. MCS diet group were received MCS diet during the whole experiment. MCD diet group were received the MCD diet during the whole experiment

### QSHY formula

QSHY formula was provided by Shanghai Sunrise Traditional Chines Medicine Co. Ltd., Shanghai, China. The powder of the formula was dissolved in water freshly and kept in 50℃water bath before gavage. Three dosages of QSHY formula (Low-dosage group: 0.29 g/kg, Medium-dosage group: 0.57 g/kg, High-dosage group: 1.14 g/kg) were gavage to rats every day. Pre-treatment group were also given medium dosage of QSHY formula (0.57 g/kg) daily.

### LC–Q-TOF–MS total ion chromatograms of QSHY formula

Mobile Phase: 0.1% aqueous formic acid (phase A) and acetonitrile (phase B), Multi-step Gradient Profile: 0–13 min, 7–43%B; 13- 15 min, 43–63%B; 15–19 min, 63–75% at a flow-rate of 0.4 mL/min.MS was operated in the positive ion mode with the acquisition rate of 0.2 s/scan. ESI source were set as follows: capillary voltage of 4.0 kV, sample cone voltage of 80 V, source temperature of 150 ℃, desolvation temperature of 500 °C at a nitrogen gas flow of 900 L/h, and cone gas flow of 50 L/h. A Leucine-enkephalin Calibrant Solution (20 ng/mL).

### Biochemical analysis

Blood were collected from tail vein into 1.5 ml EP tube every two weeks after fasting overnight. Then the blood were centrifuged at 3000 rpm at 4 °C. Alanine aminotransferase (ALT), aspartate aminotransferase (AST), total cholesterol (TCHO) and triglycerides (TG) were detected to show the levels of liver injury.

### Histopathological analysis

Steatohepatitis was observed by histopathological section. In all experiment group, 6-μm-thick sections of the liver samples which were fixed by 4% Paraformaldehyde (PFA) and embedded by paraffin, were due to hematoxylin and eosin staining (H&E) for analyzing the degree of inflammation and hepatic steatosis.

### mRNA extraction and real-time Polymerase Chain Reaction (RT-PCR)

Livers were weighted in 2 mL tube and TRIZOL (Invitrogen, 100 mg: 100 mL) were added into tubes. RNA extraction protocols followed by the instruction. RNA concentration and quality were measure by Nanodrop. 2 μg RNA were used in cDNA synthesize using a cDNA Reverse Transcription Kit. The primer for qPCR was shown in Table 2. The threshold cycle (Ct), the cycle number at which the amounts of amplified genes of interest reached a fixed threshold, was determined. Relative expression of the RT-PCR product was calculated by using the comparative $2^{{ - \Delta \Delta {\text{C}}_{{\text{t}}} }}$ method. The endogenous control β-actin was used for normalization.

**Table 2:** Primer information

Target genes	Forward sequence 5′-3′	Reverse sequence 5′-3′
PPAR-γ	CCCTGGCAAAGCATTTGTAT	ACTGGCACCCTTGAAAAATG)
β-actin	AGCCATGTACGTAGCCATCC	CTCTCAGCTGTGGGTGGTGAA
FOXA3	GACTCATGCCAAACCACCTT	TCATTGAAGGACAGCGAGTG
HNF1A	CAGCCACAACCATTCACATC	GCCATCTGGGTGGATAAA
HNF4A	AAATGTGCAGGTGTTGACCA	CACGCTCCTCCTGAAGAATC

cDNA and mRNA sequences were searched from KEGG or NCBI and primers were design by the NCBI primer designer

### Western blotting

Protein expression was detected and measured by Western blotting assays. The liver samples were homogenized with RIPA containing phosphate and protease inhibitors using a homogenizer for 5 min and then incubated for 20 min on ice. Then the lysed samples were centrifuged at 12,000 g. The supernatant was collected and protein concentration was measured with Invitrogen BCA Protein Assay Kit. Proteins were denatured by boiling at 99 °C for 5 min with loading buffer. The denatured proteins were separated by using 12% SDS-PAGE. After that, proteins were transfer from the gel to polyvinylidene fluoride membranes. 5% milk diluted with TBST buffer was used to block the nonspecific binding site for 1 h at room temperature. Primary antibodies were then applied and incubated at 4℃ overnight. After incubation, the membranes were washed three times for 5 min each with TBST buffer and then incubated with appropriate HRP-conjugated secondary antibody for 60 min at room temperature. The protein bands were visualized with chemiluminescent reagents and quantified using Image J software. β-actin was used as reference protein. Nuclear protein extraction protocols followed the guide of Beibo company which were used in our research.

### Statistical analysis

Data was presented as means ± SD. One-way ANOVA followed by Kruskal–Wallis’s multiple comparison tests were used to compare the differences between the groups. *P-*value < 0.05 was considered as statistically significant.

## Results

### Bodyweight, ratio of liver weight to bodyweight, Serum level of TC, TG, ALT and AST

**Fig.1:**
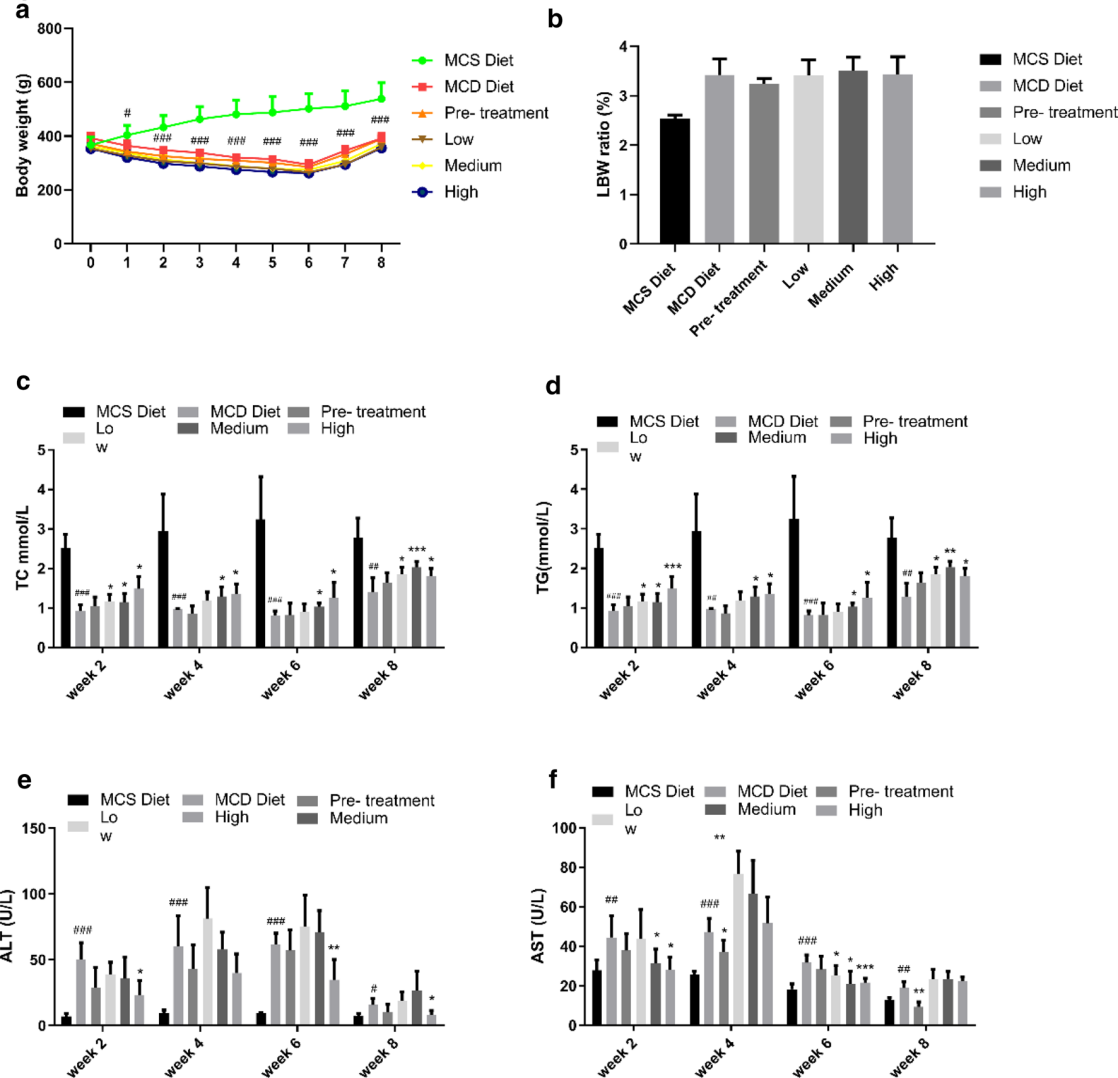
**a** Body weight. Body weight every week (n = 6 per group). **b** The ratio of liver weight to body weight (n = 6 per group). Livers were isolated and washed with 0.9% saline at the end of the experiment. Liver weight were measured and the ratio of liver weight to body weight were calculated. **c** Serum level of total cholesterol (TCHO) (n = 6 per group). Blood were collected every two weeks after 16 h fasting by cutting the tail vein. Serum were collected after centrifuged at 3000 rpm at 4 ℃. TCHO were measured using a kit. **d** Serum level of triglyceride (TG) (n = 6 per group). Using a TG testing kit to determine the serum level of triglyceride. **e** Serum level of ALT (n = 6 per group). **f** Serum level of AST (n = 6 per group). Serum level of ALT and AST were measured using kits. Data are expressed as mean ± SD.*P < 0.05 vs MCD diet, **P < 0.01 vs MCD diet, ***P < 0.05 vs MCD diet, #P < 0.05 vs MCS diet, ##P < 0.01vs MCD diet

To investigate the effect of QSHY formula on body composition and lipid metabolism, bodyweight, liver weight and serum level of TC, TG, ALT and AST were measured. The body weight (Fig. 1a) and the ratio of liver weight to body weight (Fig. 1b) showed no significant changes among the 3 treatment group. An increase in the serum level of TC (Fig. 1c) and TG (Fig. 1d) was observed among the treatment groups. In order to assess the liver function, the serum levels of ALT and AST enzymes were determined. As shown in Fig. 1e and f, at week 2 and week 6, the serum level of ALT decreased significantly comparing with the High group with MCD diet group. At second week, the serum level of AST decreased significantly when comparing the High-dosage group with the MCD diet group as shown in Fig. 1f. However, the serum level of AST increased significantly when comparing the Low-dosage group and the Medium-dosage group with the MCD diet group at week 4 and week 8. At week 6, the serum level of AST decreased significantly in all of the 3 treatment groups when compared with the MCD diet group. ALT is a kind of chemical that the liver uses to make glycogen. AST is found in a variety of tissues, including liver, brain, pancreas, heart, kidneys, lungs, and skeletal muscles. If any of these tissues are damaged, AST will be released into the bloodstream [21, 22]. While increased AST levels are indicative of a tissue injury, it is not specific to the liver per se [23, 24]. By contrast, ALT is found primarily in the liver. Any elevation of the ALT is a direct indication of a liver injury, whether minor or severe. For the Pre-treatment group, the serum levels of both ALT and AST decreased indicating that pre-treatment was more effective than other QSHY treatment group in our research.

### QSHY formula improves liver injuries induced by MCD diet

**Fig. 2:**
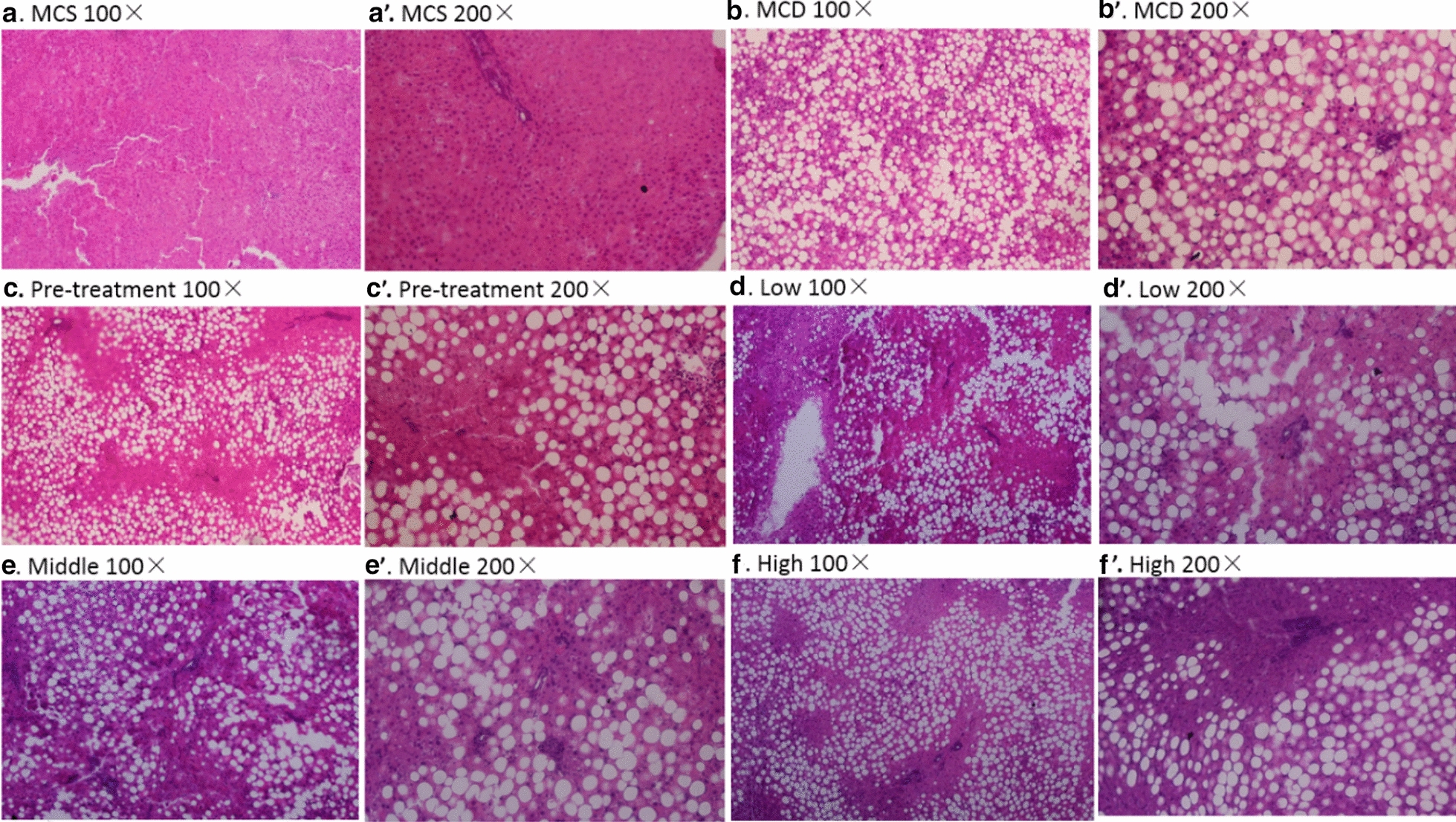
H&E staining. 6 μm liver paraffin sections were stained with H&E (**a-a’**) An example of MCS group (**b-b’**) An example of MCD group (**c–c’**) An example of pre-treatment group (**d-d’**) An example of low group (**ee’**) An example of Middle group (**f-f’**) An example of high group

Liver H&E staining was performed to evaluate liver injuries. As shown in Fig. 2 and Table 3, no pathological changes were observed in the MCS diet group and the livers appeared to be normal histologically. However, grade 5 liver steatosis and lobular inflammation were resulted from the MCD diet. Livers from the treatment groups demonstrated a lower degree of steatosis and inflammation foci. These results suggested that treatment with QSHY formula might have protected the liver from liver injuries induced by MCD diet.

**Table 3:** Summary of histopathological lesions in the liver

Group	Steatosis	Inflammatory cells
MCS Diet	0 ± 0	0 ± 0
MCD Diet	4 ± 0^###^	2.29 ± 0.49^###^
Pre-treatment	3 ± 0.53***	1 ± 0.53***
Low	2.875 ± 0.33***	1.38 ± 0.52**
Middle	2.75 ± 0.46***	1 ± 0.53***
High	2.625 ± 0.52***	0.25 ± 0.46***

Means ± SD are shown (*P < 0.05 vs MCD diet, **P < 0.01 MCD diet, ***P < 0.001 MCD diet, ^###^P < 0.001 vs MCS diet)

These values are averaged from the grading score of steatosis, which was graded 0–4 based on the average percent of fat-accumulated hepatocytes per field at × 100 magnification under H&E staining (Grading 0 =  < 5%, 1 = 5 ~ 25%, 2 = 26 ~ 50%, 3 = 51 ~ 75%, 4 =  > 75%)

Overall assessment of all inflammatory foci per field at × 200 magnification under H&E staining, which was graded 0–3 (Grading 0 = 0, 1 =  < 2, 2 = 2–4, 3 =  > 4)

### QSHY formula decreased FAS protein expression without effects on SREBP-1c protein expression

**Fig. 3:**
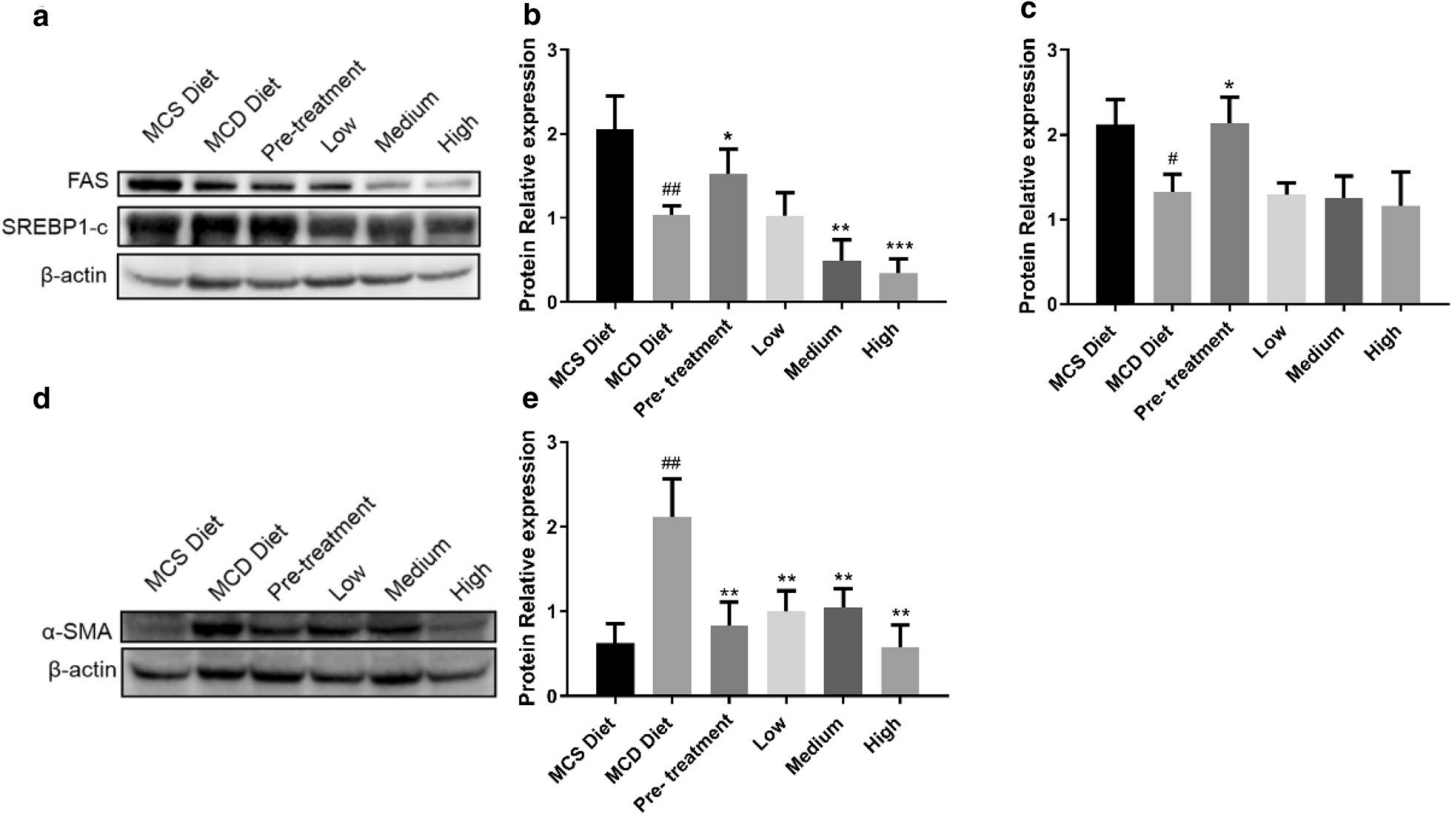
FAS, SREBP1-c and α-SMA protein expression level. Protein were extracted from livers and analysed by western blotting. Image J was used to do quantitative analysis. The lysate sample of each group was a mixture of 6 mice from the same group (n = 6 per group). The mixture sample of each group was prepared 3 times and the experiments were repeated at least 3 times. β-actin was used as a loading control **a** β-actin, FAS and SREBP1-c protein expression. **b** Quantity of FAS relative protein expression to β-actin. **c** Quantity of SREBP1-c relative protein expression to β-actin. **d** β-actin and α-SMA protein expression. **e** Quantity of α-SMA relative protein expression to β-actin. Data are expressed as mean ± SD. P value < 0.05 were considered significant. *P < 0.05 vs MCD diet, **P < 0.01 vs MCD diet, ***P < 0.05 vs MCD diet, #P < 0.05 vs MCS diet, ##P < 0.01vs MCD diet

To investigate the effect of QSHY formula on lipid synthesis, FAS and SREBP-1c protein expression were measured. Fatty acid synthase (FAS) and Sterol regulatory element-binding transcription factor 1 (SREBP-1c) are critical enzymes in lipid synthesis. As shown in Fig. 3, significant changes in SREBP-1c expression were observed in the Pre-treatment group but not the 3 treatment groups, and obvious changes in FAS expression were observed in all treatment groups. SREBP-1c not only transduces the insulin signal but is also involved in the hepatic steatosis. In our research, the QSHY formula only affected FAS protein expression without changing SREBP-1c protein expression.

### QSHY formula decreased α-SMA protein expression

To investigate the effect of QSHY formula on liver fibrosis, the protein expression level of α-smooth muscle actin (α-SMA) was detected. MCD diet can induce liver inflammation which is the main cause of liver fibrosis. α-SMA is a biomarker for tissue fibrosis. Results of western blotting analysis for α-SMA were shown in Fig. 3d. MCD diet increased the expression level of α-SMA while the QSHY formula decreased α-SMA protein expression significantly.

### The effect of QSHY formula on MAPKs pathway

**Fig. 4:**
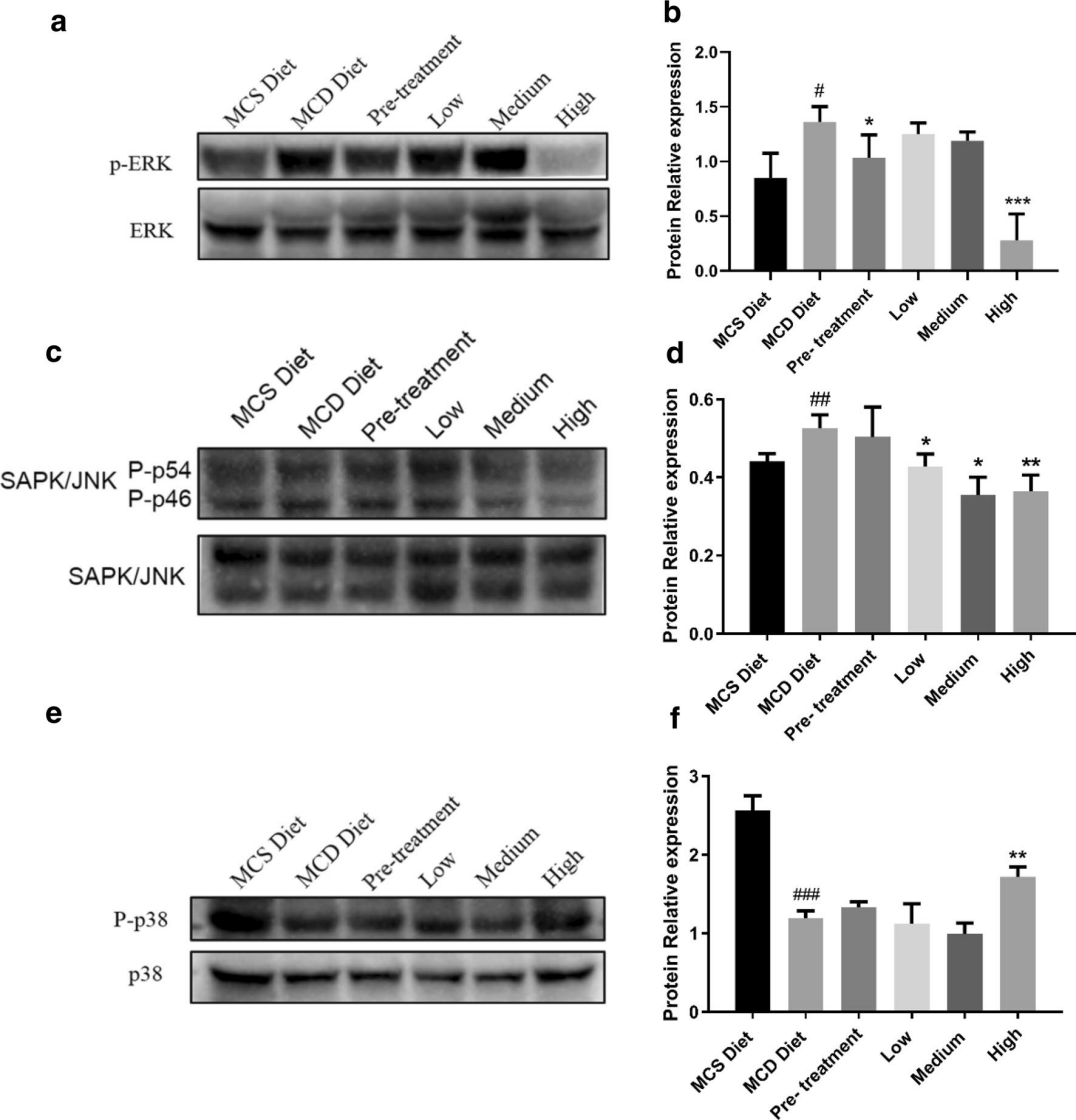
Protein expression level of MAPKs pathway. Protein were extracted from livers and western blotting was used to analysis protein expression level. The lysate sample of each group was a mixture of 6 mice from the same group (n = 6 per group). The mixture sample of each group was prepared 3 times and the experiments were repeated at least 3 times. β-actin was used as a loading control **a** p-ERK and ERK protein expression level. **b** Quantity of p-ERK protein relative expression to ERK. **c** p-p54/p-p46 SAPK/JNK and SAPK/JNK protein expression. **d** Quantity of p-p54/p-p46 SAPK/JNK relative protein expression to SAPK/JNK. **e **p-p38 and p38 protein expression. **f** Quantity of p-p38 relative protein expression to p38. Data are expressed as mean ± SD. P value < 0.05 were considered significant.*P < 0.05 vs MCD diet, **P < 0.05 vs MCD diet, ***P < 0.05 vs MCD diet, #P < 0.05 vs MCS diet, ##P < 0.01 vs MCS diet

To investigate the potential mechanism of QSHY formula on NASH and NAFLD, MAPKs pathway including p38, ERK and JNK were measured and the results were shown in Fig. 4. Only High-dosage group showed strong effect on ERK and JNK phosphorylation and enhancing p38 phosphorylation.

### QSHY formula affected PPAR-γ and p-p65 translocated into nucleus

**Fig. 5:**
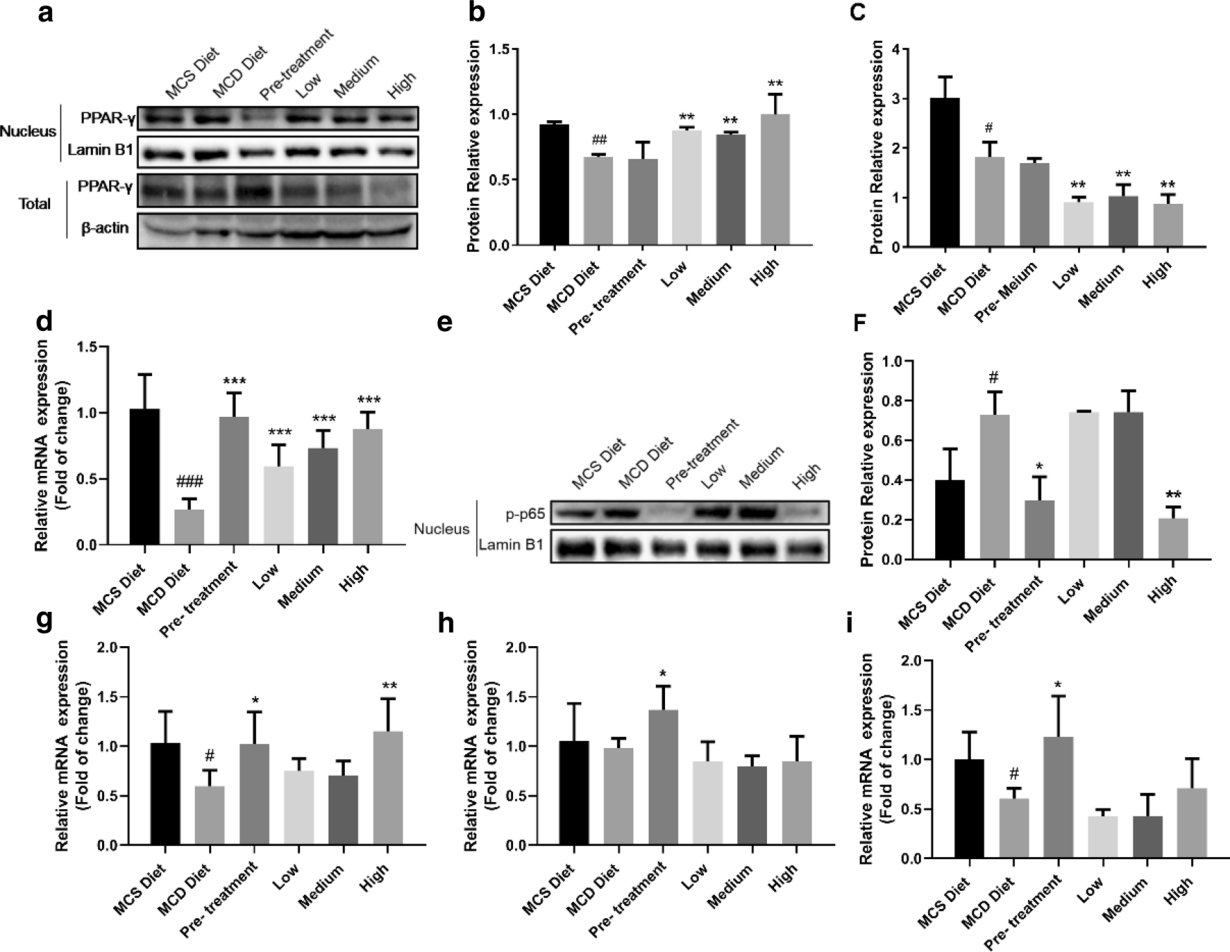
The potential mechanism of QSHY formula on inflammation and HSCs reprogramming. Protein and RNA were extracted from livers, western blotting and real-time PCR were used to analysis protein and mRNA expression. Nucleus protein extraction kit was used to analysis protein expression level in nucleus. The lysate sample of each group was a mixture of 6 mice from the same group (n = 6 per group). The mixture sample of each group was prepared 3 times and the experiments were repeated at least 3 times. β-actin was used as a loading control **a** PPRA-γ protein expression in nucleus, Lamin B1 protein expression, PPRA-γ protein expression in whole cell and β-actin protein expression. **b** Quantity of PPRA-γ protein relative expression in nucleus to Lambin B1. **c** Quantity of PPRA-γ protein relative expression in whole cell to β-actin. **d** PPRA-γ relative mRNA expression. **e** p-p65 and Lamin B1 protein expression in nucleus. **f** Quantity of p-p65 relative protein expression to Lamin B1. **g** HNF4A relative mRNA expression. **h** FOXA3 relative mRNA expression. **i** HNF1A relative mRNA expression Data are expressed as mean ± SD. P value < 0.05 were considered significant.*P < 0.05 vs MCD diet, **P < 0.05 vs MCD diet,***P < 0.05 vs MCD diet, #P < 0.05 vs MCS diet, ##P < 0.01 vs MCS diet, ###P < 0.001 vs MCS diet

To investigate the effect of QSHY formula on inflammation related pathway, PPAAR-γ, p65 and phos-p65 were measured. The expression level of PPAR-γ in mRNA and protein were both measured (Fig. 5). Real-time PCR analysis showed that QSHY formula increased PPAR-γ mRNA expression level comparing with MCD group (Fig. 5d). QSHY treatment increased the nuclear translocation of PPAR-γ and decreased protein expression level of PPAR-γ in whole cell relatively with QSHY treatment (Fig. 5a–c). These results indicated that the QSHY formula could enhance PPAR-γ mRNA expression and translocate into nucleus. Treatment with the MCD diet increased the nuclear translocation of phosphorylated p65, which was significantly inhibited in both the Pre-treatment and the High-dosage groups but not so in the Low-dosage and the Medium-dosage groups. The data suggested the inhibitory effect of QSHY on NF-kappa B signalling pathway.

### The effect of QSHY formula on HNF1A, HNF4A and FOXA3 mRNA expression.

To investigate the effect of QSHY formula on liver fibrosis, HSCs reprogramming related gene expression level was measured. HNF1A, HNF4A and FOXA3 can reprogram myofibroblasts derived from primary hepatic stellate cells into hepatocytes [25]. The mRNA expression of these three genes had been tested using Real-time PCR (Fig. 5g–i). The mRNA expression of the three genes was enhanced in the Pre-treatment group but in the High-dosage group, only the gene HNF4A exhibited enhanced mRNA expression. All these results suggested that QSHY formula improved liver fibrosis partly by enhancing HSCs reprogramming.

### Positive or tentative identification of QSHY formula

The tentative identification of bioactive compounds in the extract of QSHY formula was carried out using positive ionization of Liquid chromatography-mass spectrometry quadrupole time of flight (LC-Q-TOF/MS). Table 4 illustrates the LC-Q-TOF/MS base peak intensity profiles QSHY formula. The top one compounds is Geniposide.

**Table 4:** Positive or tentative identification of QSHY formula

Peak no.	Retention time (min)	m/z	Positive mode	MS/MS fragments	Molecular formula	Structural identification
1	3.377	355.1027	M + H	163.0325	C16H18O9	Heriguard
2	4.014	551.2018	M + H	249.0669,209.0750,149.0571	C23H34O15	Genipin 1-gentiobioside
3	4.814	389.1412	M + H	209.0750,149.0571	C17H24O10	Geniposide
4	6.454	391.1325	M + H	229.0827,207.0604	C20H22O8	Polydatin
5	8.192	499.1188	M + H	369.1129,207.0604	C28H34O8	Uliginosin B
6	8.277	603.165	M + H	588.4108	C36H42O8	Sarothralin G
7	8.872	449.102	M + H	303.0477	C21H20O11	Quercetin-7-O-rhamnoside
8	9.317	411.1156	M + H	229.0827,163.0325	C29H48O	Stigmasterol
9	9.666	679.5173	M + H	340.2598		Unknown
10	9.866	697.2432	M + H	471.1478,309.0950,147.0392	C32H40O17	6′-O-P-Coumaroylgenipin gentiobioside
11	10.66	792.5974	M + H	396.798		Unknown
12	10.929	977.3885	M + H	675.2690,485.1959,329.1692	C44H64O24	Crocin
13	11.277	275.1606	M + Na	217.1535,171.1445	C15H24O3	Zedoarondiol
14	12.223	409.1422	M + H	247.0940,229.0827	C20H24O9	Torachrysone-8-O-beta-D-glucoside
15	13.066	455.0968	M + H	271.0566	C23H18O10	kaempferol tetraacetate
16	14.558	469.1132	M + NH4	285.0727,246.2372	C21H20O10	Anthraglycoside A

The LC-Q-TOF/MS base peak intensity profiles QSHY formula

## Discussion

NAFLD and NASH are growing health problems across the world complicated with other common comorbidities such as obesity, type II diabetes, hyperglycemia, and so on. NAFLD and NASH are characterized with severe inflammation and steaohepatitis and there is currently no established therapy for NASH. TCM, due to their multiply active compounds and multiple drug targets, have shown great potential advantages for curing NASH.

In this study, a MCD diet induced NASH model experiment was conducted to evaluate the effects of the QSHY formula. According to the findings about the serum level of TC, TG, ALT, AST and H&E staining in this study, although the rats received MCD diet for the first 6 weeks and the MCS diet in the last two weeks, hepatic steatosis and inflammation were not severe. Therefore, considering the limitation of the MCD model of inhibiting the lipid exporting from the liver, a mixed-diet of MCS: MCD with the ratio of 1:1 was applied in the last two weeks.

It was observed that the serum levels of ALT and AST, hepatic fat content, size of lipid droplet, the number of inflammation foci and α-SMA expression were reduced with the treatment of the QSHY formula. Based on the study findings, some probable mechanisms were further discussed in the following.

FAS catalyzes the synthesis of long-chain fatty acids from acetyl-CoA and malonyl-CoA [26]. FAS is responsible for producing lipids in the liver and exporting to metabolically active tissues or storage in adipose tissue while the MCD diet can block the lipid exportation [27]. This may explain why all rats received MCD diet showed lower expression of FAS. However. after changing MCD diet to a combination of MCS & MCD diet, QSHY formula (except Pre-treatment group) decreased FAS expression that may be due to the suppressed lipid synthesis in liver. For the Pre-treatment group, QSHY formula enhanced FAS expression possibly because the rats were in different progression of NAFLD compared with other QSHY treatment groups. After changing the MCD diet to the combination of MCS and MCD diet, QSHY formula enhanced FAS expression that may partly explain the reduced lipid synthesis in liver. Increased interest has focused on FAS as a potential target for the diagnosis and treatment of metabolic syndrome [28, 29].

α-SMA positive cells can be found in the vascular walls of the central vein and portal tract but they also appear in fibrotic area. α-SMA is a good marker of myofibroblast and its appearance in liver mesenchymal cells seems closely related to the process of hepatic fibrosis[30]. Hepatic fibrosis is a symbol of NASH and the QSHY formula could inhibit hepatic α-SMA protein expression suggesting its anti-fibrosis effect. Chronic inflammation is a major cause for most fibrosis including liver fibrosis [31, 32]. The mitogen-activated protein kinase (MAPK) signaling pathways (ERKs: extracellular-signal-regulated kinases; JNKs: Jun-amino-terminal kinases; P38/SAPKs: stress-activated protein kinases) are activated by a variety of extra and intracellular stimuli including cytokines, growth factors, and hormones. These pathways play critical roles in the regulation of many cellular processes including proliferation, differentiation, the stress response, motility, growth, differentiation, survival, and death. In this study, it was shown that the QSHY formula suppressed ERK and JNK phosphorylation. An excess of non-ester fatty acids (NEFAs) existed lipotoxicity induced excessive ROS which overactivated the hepatic JNK and p38MAPK pathway and then impaired insulin signaling pathway in patients with NASH [33]. QSHY formula therapy in our research effectively suppressed ERK and JNK phosphorylation that may partly explain the possible mechanism of its hepatoprotective effect.

Transcription factors of the nuclear factor κB (NF-κB)/Rel (p65) family play an important role in inflammatory and immune responses [34]. After NF-κB translocating into nuclear, its downstream genes expression such as IL-6, TNF-α, MCP1 will be increased. Detecting the phosphorylated p65 (p-p65) in nuclear is widely used to investigate whether the NF-κB pathway is activated. QSHY formula suppressed p-p65 translocated into nuclear obviously suggesting that the effect of QSHY formula on NASH may be partly mediated via suppressing NF-κB pathway activation.

Peroxisome proliferator-activated receptor-gamma (PPAR-γ) is a transcription factor which regulates lipid metabolism and inflammatory responses. PPAR-γ deficient (PPAR-γ ±)mice fed the MCD diet developed more severe steatohepatitis than wild-type mice and PPARγ activation suppressed hepatic lipoperoxidation [35]. The content of PPAR-γ in nucleus was obviously increased in the QSHY formula treatment groups indicating that QSHY formula may enhance PRAR-γ nuclear translocation to improve NASH in our research.

Hepatic stellate cells (HSCs) are resident mesenchymal cells that keep features of resident fibroblasts and pericytes and comprise 15% of the total resident cells in normal human liver. Quiescent HSCs are activated and then transdifferentiate into proliferative, migratory, and contractile myofibroblasts, manifesting pro-fibrogenic transcriptional and secretory properties. This process can be reversed and forkhead box protein A3 (FOXA3), transcription Factor 1 (HNF1A), Hepatocyte Nuclear Factor 4 alpha (HNF4A) play important roles in HSCs reprogramming process [36]. QSHY formula enhanced these three genes mRNA expression indicating the anti-fibrosis effect of QSHY formula may be involved reinforced HSCs reprogramming.

**Fig. 6:**
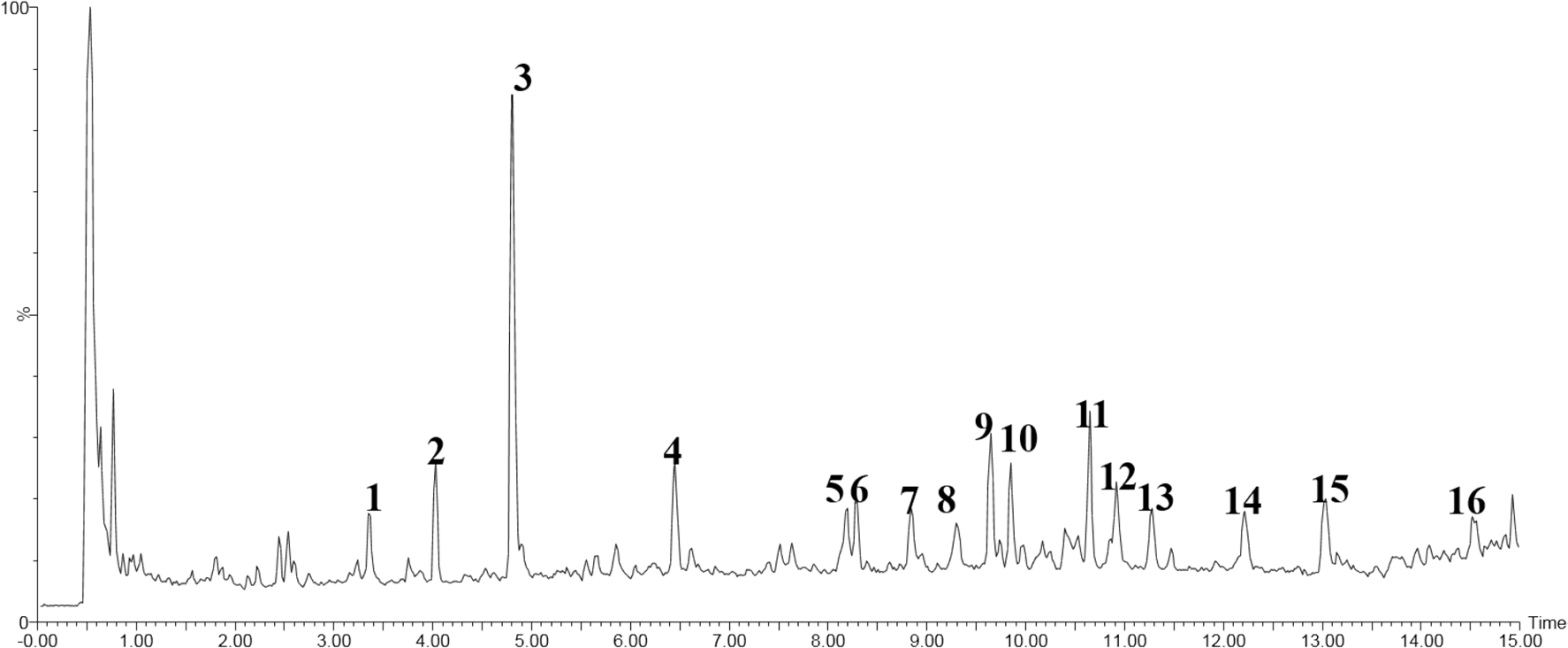
LC–Q-TOF–MS total ion chromatograms of QSHY formula. The tentative identification of bioactive compounds in the extract of QSHY formula was carried out using positive ionization

The most abundant ingredient in QSHY formula is Heriguard as shown in Fig. 6 and Table 4. Heriguard is one of the most available acids which can be easily found in green coffee extracts and tea. Heriguard has antioxidant activity, antibacterial, hepatoprotective, anti-obesity and anti-hypertension [37]. The most important bioactivity of Heriguard is improving metabolic syndrome by regulating PPAR-γ [38] and MAPK pathways [43].

The therapeutic mechanism of QSHY formula on MCD diet induced liver injuries found in our research is novel. We observed QSHY formula can improve α-SMA expression and regulate HSCs reprogramming genes expression. These results mean QSHY formula can improve early stage of fibrosis induced by the MCD diet by regulating HSCs reprogramming.

## Conclusion

These findings suggest that the QSHY formula exerts a hepatoprotective effect against steatosis and fibrosis presumably via depressed MAPK pathways phosphorylation, reinforcement of PPAR-γ and p-p65 translocating into nucleus and enhanced HSCs reprogramming. These pathways perform a probable therapeutic target for steatosis and NASH in drug development (Additional file 1).

## Supplementary Information

**Additional file 1**.

## Data Availability

Yes.

## Abbreviations

ANOVA: Analysis of variance
ALT: Alanine aminotransferase
AST: Aspartate aminotransferase
BMI: Body Mass Index
Ct: Threshold cycle
EMG: Electromyography
ERK: Extracellular-signal-regulated kinases
FASN: Fatty Acid Synthase coding gene
FOXA3: Forkhead box protein A3
HNF1A: Transcription Factor 1 alpha
HNF4A: Hepatocyte Nuclear Factor 4 alpha
HSCs: Hepatic stellate cells
JNK: Jun-amino-terminal kinases
MAPK: Mitogen-activated protein kinase
MCD: Methionine-choline-deficient diet
MCS: Methionine-choline- supplemented diet
NAFLD: Nonalcoholic fatty liver diseases
NASH: Non-alcoholic steatohepatitis
NF-κB: Nuclear factor κB
OCT: Optimal cutting temperature compound
p38: P38 mitogen-activated protein kinases
p-p65: Phosphorylated p65
PPAR-γ: Peroxisome proliferator-activated receptor-gamma
PGC-1α: Peroxisome proliferator-activated receptor gamma co-activator 1-alpha
qPCR: Quantitative polymerase chain reaction
QSHY: Qushihuayu
RT-PCR: Real-time polymerase chain reaction
SD.: Standard Deviation
SREBP1-c: Sterol regulatory element binding transcription factor 1 c
TCM: Traditional Chinese Medicine
TCHO: Total cholesterol and
TG: Total triglycerides
